# Solid-state NMR analysis of unlabeled fungal cell walls from *Aspergillus* and *Candida* species

**DOI:** 10.1016/j.yjsbx.2022.100070

**Published:** 2022-07-19

**Authors:** Liyanage D. Fernando, Malitha C. Dickwella Widanage, S. Chandra Shekar, Frederic Mentink-Vigier, Ping Wang, Sungsool Wi, Tuo Wang

**Affiliations:** a Department of Chemistry, Louisiana State University, Baton Rouge, LA 70803, USA; b National High Magnetic Field Laboratory, Tallahassee, FL 32310, USA; c Department of Microbiology, Immunology, and Parasitology, Louisiana State University Health Sciences Center, New Orleans, LA 70112, USA

**Keywords:** Solid-state NMR, Dynamic nuclear polarization, *Aspergillus fumigatus*, *Candida albicans*, Polysaccharides, Cell walls

## Abstract

Fungal infections cause high mortality in immunocompromised individuals, which has emerged as a significant threat to human health. The efforts devoted to the development of antifungal agents targeting the cell wall polysaccharides have been hindered by our incomplete picture of the assembly and remodeling of fungal cell walls. High-resolution solid-state nuclear magnetic resonance (ss NMR) studies have substantially revised our understanding of the polymorphic structure of polysaccharides and the nanoscale organization of cell walls in *Aspergillus fumigatus* and multiple other fungi. However, this approach requires ^13^C/^15^N-enrichment of the sample being studied, severely restricting its application. Here we employ the dynamic nuclear polarization (DNP) technique to compare the unlabeled cell wall materials of *A. fumigatus* and *C. albicans* prepared using both liquid and solid media. For each fungus, we have identified a highly conserved carbohydrate core for the cell walls of conidia and mycelia, and from liquid and solid cultures. Using samples prepared in different media, the recently identified function of α-glucan, which packs with chitin to form the mechanical centers, has been confirmed through conventional ss NMR measurements of polymer dynamics. These timely efforts not only validate the structural principles recently discovered for *A. fumigatus* cell walls in different morphological stages, but also open up the possibility of extending the current investigation to other fungal materials and cellular systems that are challenging to label.

## Highlights

• An NMR investigation strategy with atomic resolution for unlabeled fungal cell walls.
• Conserved carbohydrate core revealed in conidia and mycelia of *Aspergillus fumigatus.*
• Confirmation of the structural function of α-glucans in *A. fumigatus.*
• Carbohydrate fingerprints preserved in liquid and solid cultures of *Candida albicans.*

## Graphical abstract

**Figure:**
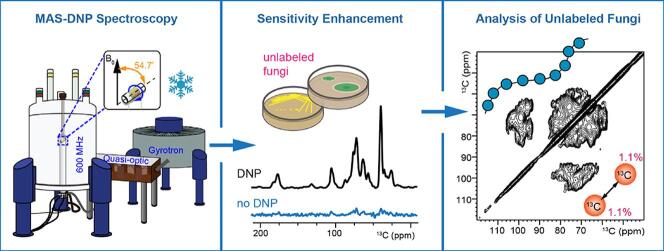

## Introduction

The carbohydrate components in the fungal cell walls are promising therapeutic targets of novel antifungal drugs for combating life-threatening infections by pathogenic fungi (Brown, 2012). Two families of compounds (echinocandins and terpenoids) have been developed to disrupt the biosynthesis of β-glucans in the cell wall, and another antifungal named nikkomycin is a potent inhibitor of chitin synthesis (Perlin, 2015, Denning, 2003, Jallow and Govender, 2021, Li et al., 2019, Perlin, 2020). Unfortunately, nikkomycin only has weak activity against most fungal species, and the β-glucan inhibitors often suffer from the compensatory paradoxical effect, through which the chitin level is massively elevated to compensate for the loss of β-glucans (Aruanno et al., 2019, Loiko and Wagener, 2017). Hence, an in-depth understanding of the biosynthesis and structure of fungal polysaccharides, as well as the structural dynamics of the assembled cell walls, may facilitate the discovery of more effective antifungal compounds from the structural perspective.

Solid-state NMR (ss NMR) spectroscopy is a non-destructive and high-resolution method for elucidating the structure of fungal biopolymers (Ghassemi, 2022). This method has been extensively employed to track melanization in *Cryptococcus neoformans* and other fungi (Zhong et al., 2008, Chatterjee et al., 2015, Chrissian, 2020). Studies have also been conducted on the analysis of *Aspergillus fumigatus* biofilm, with a focus on the quantification of carbon contributions in the extracellular matrix (Reichhardt, 2015, Reichhardt et al., 2019). Additionally, multidimensional ss NMR methods have been employed to investigate the nanoscale organization of *A. fumigatus* cell walls as well as its responses to stresses (Kang, 2018, Chakraborty, 2021). The samples being used in these studies are intact cells without chemical treatment, which allowed the investigation of biomolecules in a fully native physical state. This method was also combined with chromatography and mass spectrometry to understand the cell wall structure of *Schizophyllum commune* (Ehren, 2020).

Notably, in two recent ss NMR studies of *A. fumigatus*, a substantially revised structural scheme of the mycelial cell wall has been proposed (Kang, 2018, Chakraborty, 2021). The rigid and hydrophobic scaffold of the *A. fumigatus* cell wall is formed by tightly associated chitin and α-1,3-glucan, which is dispersed in a mobile and hydrated matrix formed by branched β-1,3-glucans. Two other polysaccharides, including galactomannan and galactosaminogalactan, are found to mainly form the outermost dynamic layer, together with proteins on the cell surface. These structural aspects derived from ss NMR data provide a physical vision of polymer dynamics and packing, complementing the existing chemical analyses that reveal a chemical vision of the polymer cross-linking and extractability. Functional genomics approaches were also employed to generate mutant lines, each of which selectively remove an important carbohydrate component, to evaluate the functions of different polysaccharides and the remodeling of cell walls under internal stresses (Chakraborty, 2021). Statistical methods are also available for analyzing the polymorphic structure of polysaccharides in living fungal cells as demonstrated using chitin and chitosan (Fernando, 2021).

These ss NMR studies often require isotopic enrichment of the sample, e.g., by ^13^C and ^15^N, to allow 2D and 3D correlation experiments to be completed in a reasonable time frame, yet with adequate resolution. In fungal research, this has been achieved by germinating conidia into mycelia in minimal liquid media containing ^13^C-glucose and ^15^N sources. But there is a dire need for a method to validate the recent structural findings by examining samples prepared in solid media, a condition more widely used in microbiology research, in order to better integrate the structural concepts derived from ss NMR and biochemical approaches to promote sporulation (Chakraborty, 2021, Latgé and Wang, 2022). Also, the previous studies of *A. fumigatus* were primarily focused on the mycelium. It is questionable if the structural concepts could be applied to cells at different developmental stages, for example, the conidium. The inhaled conidia *of A. fumigatus* germinate into vegetative hyphae, invading the patient lungs during infection (Dagenais and Keller, 2009, van de Veerdonk et al., 2017). Even though it has been shown that the conidial and mycelial cell walls of *A. fumigatus* differ in their composition and organization (Latgé et al., 2017), at this moment, we still lack a detailed molecular-level understanding of the conidial cell walls (Ibrahim-Granet, 2003).

Here we show that such challenges can be addressed by introducing natural-abundance (NA) magic-angle spinning dynamic nuclear polarization (MAS-DNP), which has been widely used in biopolymer and material research (Smith et al., 2019, Märker, 2017, Takahashi, 2012, Viger-Gravel, 2019, Perras, 2017), to better characterize fungal cell walls. This sensitivity-enhancing technique has made it possible to collect 2D correlation spectra using unlabeled samples. The NMR linewidths of most fungal carbohydrates are found to be substantially broadened at the cryogenic temperature of MAS-DNP due to their highly dynamic nature. A reserved carbohydrate core has been identified in *A. fumigatus* mycelia and conidia using unlabeled samples prepared in solid and liquid media, which could not be possible without the atomic resolution provided by the DNP-enabled 2D ^13^C–^13^C correlation spectra of these unlabeled materials. Further examination of polymer dynamics using conventional ss NMR at ambient temperature confirmed the recently proposed structural function of α-1,3-glucan in forming the stiff core of *A. fumigatus* cell walls in both mycelia and conidia. Finally, we extend this study to another prevalent fungal pathogen *Candida albicans*, demonstrating the applicability of these approaches in fungal research.

## Materials and methods

### Preparation of fungal materials

Four unlabeled samples from *A. fumigatus* (RL 578) and *C. albicans* (SC5314) were grown using both liquid and solid media for MAS-DNP investigations. Unlabeled *A. fumigatus* materials were prepared in two ways using either solid or liquid media. The solid culture was prepared in YPD (Yeast-Extract Peptone Dextrose) agar. The liquid cultures were prepared in 100 mL of modified minimal media containing 10.0 g/L of glucose and 6.0 g/L of sodium nitrate. The pH of the media was adjusted to 6.5. Both liquid and solid cultures were incubated for 3 days at 30 °C (at 210 rpm). Similarly, both solid and liquid cultures were prepared for *C. albicans* without labeling. The solid culture was grown in YNB (Yeast Nitrogen Base) media with agar, 2 % of glucose, and 1 % of ammonium sulfate. The liquid culture was prepared using YNB, 2 % of glucose and 1 % of ammonium sulfate with pH adjusted to 5.8–6. The fungal material was harvested by centrifugation at 7000 g for 20 min. The fungal material was washed using 10 mM phosphate buffer saline (pH 7.4) to remove excess ions. Only the greenish center region of the solid culture was collected.

To validate the results obtained on unlabeled materials described above, we also prepared ^13^C, ^15^N-labeled samples using liquid media. The uniformly ^13^C,^15^N-labeled *A. fumigatus* sample was prepared by adding 10.0 g/L of ^13^C glucose and 6.0 g/L of ^15^N-labeled sodium nitrate to the minimal liquid media (Kirui et al., 2019). The uniformly ^13^C,^15^N-labeled *C. albicans* sample was prepared by adding 2 % of ^13^C glucose and 1 % of ^15^N-labeled ammonium sulfate into the YNB liquid media. Both strains were grown for 3 days at 30 °C.

## Transmission electron microscopy and scanning electron microscopy

Transmission electron microscopy (TEM) was conducted using a JEOL JEM-1400 electron microscope. The sample was placed onto a glow discharged TEM grid for several minutes. It was stained using a mixture of 2 % uranyl acetate and lead citrate solution. The cell wall thickness was measured using ImageJ software (Schneider et al., 2012) after setting the scale in accordance with known bar scales on the cell images. Scanning electron microscopy (SEM) was conducted using an FEI Quanta 3D FEG field emission scanning electron microscope to examine the surface morphology of the cells. Briefly, cells were collected by filtration and fixed on a 0.4 μm pore polycarbonate filter in 2 % glutaraldehyde, 2 % formaldehyde, and 1 % OsO4. The sample was rinsed with distilled water, dehydrated with graded ethanol series, and dried with HMDS reagent. The cells were mounted to aluminum specimen stubs and coated with platinum in an EMS550X sputter coater for imaging.

## Sample preparation for MAS-DNP measurements

Unlabeled fungal materials were mixed with the stock solution containing the biradicals needed for MAS-DNP. The stock solution contains 30 mM of AsymPol-POK biradicals (Mentink-Vigier, 2018, Harrabi, 2022) in 40 µL of d6-DMSO-D_2_O-H_2_O (1:8:1 vol%) that was used to avoid ^13^C signal contribution from the solvents (e.g., from glycerol). The volume percentages of different solvents used here deviate from conventional recipes used for MAS-DNP of biomolecular samples. It is fully based on repeated optimizations of these fungal materials for the best sensitivity. To test the effect of the DNP juice, a different solvent of ^13^C-depleted, d_8_-glycerol/D_2_O/H_2_O (6:3:1 vol%), as well as the AMUPol biradicals (Sauvée, 2013), were used for the samples prepared in solid media. The fungal samples were mildly ground using a set of mortar and pestle when being wetted by the cryoprotectant solution. This allows the radicals to penetrate and distribute in the porous cell wall, without perturbing the appearance of the fungal pellet and the molecular-level structure of molecules. Around 30 mg of fungal material was packed into 3.2 mm sapphire rotors.

## MAS-DNP experiments

The MAS-DNP experiments were conducted on a 600 MHz (14.1 T)/395 GHz instrument using a 3.2-mm HCN DNP probe. The power of microwave irradiation was around 12 W. The temperature was ∼ 100 K with microwave irradiation and decreased to 94 K when the microwave was turned off. The DNP buildup time constants were 2.6-s and 3.1-s for *C. albicans* samples prepared in solid and liquid media, respectively. The number was shortened to 1.3-s for the *A. fumigatus* solid sample. The MAS rate was 10.5 kHz for all DNP experiments unless mentioned otherwise. 1D ^1^H–^13^C cross-polarization (CP) experiments were measured using ^1^H field of 50 kHz, providing a sideband match to the ^13^C field of 39.5 kHz, and 1-ms contact time. 2D refocused dipolar (SPC5) (Hohwy et al., 1999) INADEQUATE spectra (Lesage et al., 1999) were collected under 10.5 kHz MAS. The field strength of ^1^H decoupling during SPC5 blocks was 100 kHz. A Double-Quantum Filtered (DQF) 2D dipolar ^13^C–^13^C correlation NMR experiment (Wi et al., 2022) was carried out on *A. fumigatus* solid culture. The ^1^H and ^13^C 90-degree pulse lengths were 2.5-μs and 4-μs, respectively. In the indirect dimension, 200–300 increments were collected. In total, 64, 32, and 32 transients were added for signal-averaging purposes. The DARR mixing time was either 100-ms or 250-ms. The ^1^H–^13^C CP mixing was 0.5 ms. All the acquisition parameters are summarized in Supplementary Table S1.

Spectral deconvolution was performed on 1D ^13^C CP MAS-DNP spectra of unlabeled liquid culture and solid culture of *C. albicans* to obtain the chemical shift, linewidth, and intensity of carbohydrate peaks. Spectral deconvolution was performed from 120 to 40 ppm for all the carbohydrate regions, using DmFit (Massiot, 2002). The parameters are provided in Supplementary Table S2.

## Conventional solid-state NMR experiments at room temperature

The experiments were conducted on 800 MHz (18.4 Tesla) and 400 MHz (9.4 Tesla) Brucker spectrometers equipped with 3.2 mm and 4 mm HCN probes, respectively. All experiments were conducted under 13 kHz or 13.5 kHz MAS at 298 K temperature. Approximately 30 mg of sample was packed to 3.2 mm MAS rotors and around 110 mg of sample was packed in a 4 mm ZrO_2_ rotor. The ^13^C chemical shifts were externally referenced to tetramethylsilane (TMS) scale by calibrating the Cδ peak of the Met residue in the model tri-peptide *N*-formyl-Met-Leu-Phe-OH (MLF) to 14.0 ppm. The radiofrequency field strength was 83 kHz for ^1^H decoupling and 62.5 kHz for ^13^C hard pulse and 50 kHz for ^1^H and ^13^C CP spin lock. The acquisition parameters are tabulated in Supplementary Table S1.

To compare with the results of unlabeled samples, we measured 2D ^13^C CP refocused J-INADEQUATE spectra using a uniformly ^13^C-labeled *A. fumigatus* sample (liquid culture) on the 800 MHz spectrometer. We also measured a uniformly ^13^C-labeled *C. albicans* sample (liquid culture), resulting in a 2D ^13^C direct polarization (DP) refocused J-INADEQUATE spectrum measured on the 800 MHz spectrometer and a ^13^C CP refocused dipolar (SPC5) INADEQUATE spectrum collected on the 400 MHz NMR. To investigate the polymer dynamics in the unlabeled fungal cell wall, ^13^C-*T*_1_ relaxation was measured. It was measured using CP-based *T*_1_ pulse sequences (Torchia, 1978) with a z-filter varied from 0 to 5 s. The relative intensity of each data point (relative to the first data point) was plotted as a function of time. The curve was fit using a bi-exponential equation. For ^13^C-*T*_1_ relaxation measurements, the number of scans was between 1,024 and 4,096 for each data point of *A. fumigatus* samples prepared in solid and liquid media.

## Results and discussion

### Morphological difference of fungal cells cultured in solid and liquid media

**Fig. 1:**
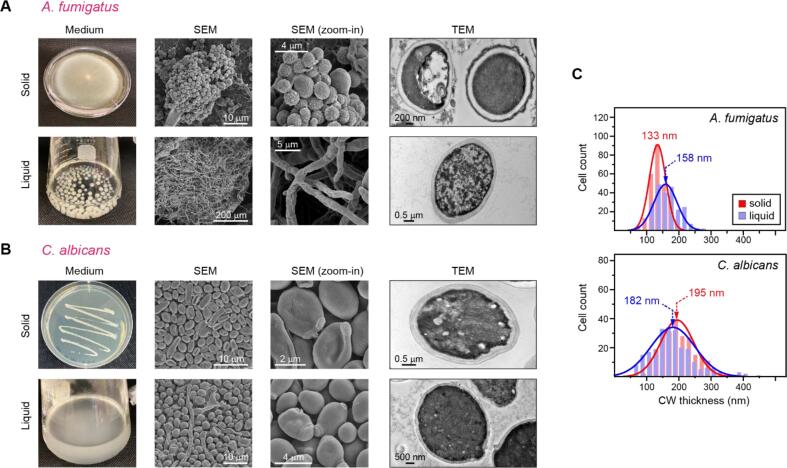
**Macro- and microscopic differences of cell morphology in 3-day-old solid and liquid cultures**. The ultrastructural features of cell walls are shown for **(A)***A. fumigatus* and **(B)***C. albicans.* From left to right, each row incorporates an image of culture, an SEM image, a zoomed-in region of the SEM image, and a TEM image. In both panels (A) and (B), the solid culture (top) and the liquid culture (bottom) are shown. **(C)** The cell wall thickness of *A. fumigatus* (top) and *C. albicans* (bottom). Data are presented as a distribution, with means of 10 measurements from 15 biological replicates of each solid or liquid culture.

*A. fumigatus* cells have different morphologies when prepared in solid and liquid media (Fig. 1A) (Bernard and Latge, 2001, Fontaine, 2010, Latgé and Chamilos, 2019, Latgé, 2017, Kang et al., 2021). In a Petri dish containing YPD agar, *A. fumigatus* exhibited circular growth with greenish-blue conidia at the center and white mycelial threads at the edge (Fig. 1A; top row). The surface morphology of the greenish-blue center region was examined using SEM images, which revealed a 2–3 μm diameter for the conidia of *A. fumigatus* (asexual spores) produced in conidiophores (fruiting body). Part of the outer cell wall of conidia should be covered with melanin, as indicated by the rough surface in the conidia observed in the zoomed-in regions of SEM images (Walker, 2010). The conidia from fungal plates were taken and inoculated into liquid minimal media and cultured at 30 °C for 3 days (Fig. 1A; bottom row). The filamentous structures observed by SEM have confirmed that *A. fumigatus* mainly grew into hyphal form under this culture condition.

Evidently, the use of solid media has promoted asexual sporulation in *A. fumigatus* samples (Fig. 1A). *A. fumigatus* adapts to stressful environments during host interactions and acquires different morphotypes in their life cycle (Gow et al., 2002, Lee and Sheppard, 2016). The composition and organization of fungal cell walls are always changing in response to the morphotypes in the life cycle and growth conditions (Garcia-Rubio et al., 2020). The mycelium is the vegetative morphotype and the conidium is typically considered the infective morphotype (Latgé, 2017). The conidia of *A. fumigatus* disperse and colonize different habitats, for instance, the lung alveoli, germinating into hyphae and causing invasive infections (Bernard and Latge, 2001, Croft et al., 2016, Abad, 2010). Therefore, it is of high significance to elucidating the cell wall structure in conidia.

However, it is not trivial to convert the solid media (for example, yeast extracts) widely used in microbiology laboratories into uniformly ^13^C,^15^N-labeled counterparts without worrying about the isotope-dilution from unlabeled components. This barrier has hindered the use of high-resolution solid-state NMR spectroscopy for characterizing fungal conidia. At the same time, fungal materials cultured in minimal liquid media could not fully represent those prepared using complex media. This situation can be improved through the development of MAS-DNP techniques, as detailed in later sections.

After 3 days of incubation in YNB solid media, *C. albicans* produced cream-colored, dull smooth yeast-like colonies (Fig. 1B; top row). SEM images revealed the oval shape of yeast-like *C. albicans* cells with diameters of 2–4 μm. In the liquid culture (Fig. 1B; bottom row), *C. albicans* cells exist as a mixture of hyphae, pseudo hypha, and yeast forms, with the yeast form being the most prominent. The hyphae and germ tubes were present as minor components and hence were excluded from further consideration. Interpretation and conclusion in later sections are drawn by treating the yeast form as the overwhelmingly dominant form in the liquid cultures.

TEM images were used to quantify the distribution of cell wall thickness (Fig. 1C and Supplementary Table S3) in *A. fumigatus* and *C. albicans* samples harvested from solid and liquid media. For *A. fumigatus*, the average thickness of the cell wall increased from 133 nm for the conidia (solid media) to 158 nm for the hypha (liquid culture). However, the change is much smaller between *C. albicans* materials prepared using solid and liquid conditions. Presently, it is not clear how the microscopic features of cellular morphology arise from the molecular-level organization of cell walls, warranting further investigations.

## Sensitivity-enhancement of fungal materials by MAS-DNP

**Fig. 2:**
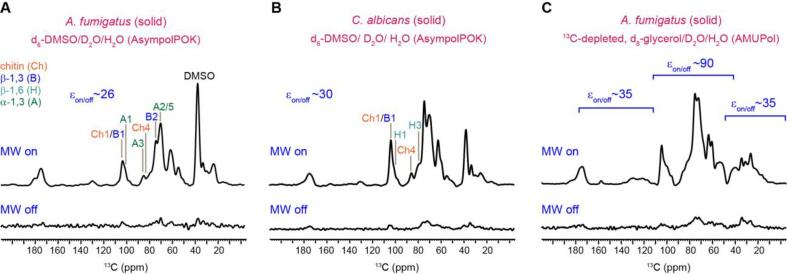
**Sensitivity enhancement by MAS-DNP on unlabeled fungal materials**. 1D ^13^C spectra of unlabeled fungal samples were shown for **(A)***A. fumigatus* solid culture (mainly conidia) and **(B)***C. albicans* solid culture (mainly yeast form) prepared using d_6_-DMSO/D_2_O/H_2_O with 20 mM AsympolPOK. The spectra collected with and without microwave (MW) radiation were compared to give the enhancement factor (ε_on/off_). **(C)** 1D ^13^C spectra of unlabeled cell walls of *A. fumigatus* solid culture (mainly conidia) in ^13^C-depleted, d_8_-glycerol/D_2_O/H_2_O matrix with 10 mM AMUPol radical. This sample has different enhancement factors for carbohydrate (ε_on/off_ ∼ 90) and protein and lipids (ε_on/off_ ∼ 35 for aliphatic, aromatic, and unsaturated carbons).

The *A. fumigatus* and *C. albicans* samples harvested from solid media were impregnated in a matrix of DMSO/D_2_O/H_2_O containing 20 mM AsympolPOK. This recently designed biradical promotes efficient polarization and provides fast DNP buildup through electron dipolar and exchange interactions (Mentink-Vigier, 2018). AsympolPOK yielded a very short DNP buildup time of 1.3–3.1 s for these fungal samples (Supplementary Table S4), making it possible to use short recycle delays, in turn resulting in the rapid acquisition of experiments. AsympolPOK biradicals were stable in fungal samples as confirmed by electron paramagnetic resonance (EPR) spectra (Supplementary Fig. 1). The sensitivity was enhanced by 26 to 30 times for both *A. fumigatus* and *C. albicans* under microwave irradiation (Fig. 2A, B), reducing the experimental time by 676–900 fold. Though the 1D ^13^C peaks were very broad, some fine features of the peaks could still be discerned. The major signals are from chitin (Ch), β-1,3-glucan (B), and α-1,3-glucan (A) in *A. fumigatus*. For *C. albicans*, the prominent peaks emanated from chitin, β-1,3-glucan, and β-1,6-glucan (H). Determination of the linewidth and analysis of the nature of these peaks were aided by 2D ^13^C correlation spectra, as described in detail later.

Notably, a record high sensitivity enhancement factor of 90-fold was achieved using an *A. fumigatus* sample doped with AMUPol using ^13^C-depleted, d_8_-glycerol/D_2_O/H_2_O solvent (Fig. 2C). The ^13^C-depleted solvent is chosen to avoid the detection of glycerol signals that overlay with carbohydrate peaks. To our best knowledge, the 90-fold enhancement is the highest value reported for any cell wall system on a 600 MHz/395 GHz DNP, but this sample was not used for measuring 2D experiments. The first reason is the prohibitively long DNP buildup time, 5.0 s for this sample instead of 1.3 s for the other *A. fumigatus* sample (Supplementary Table S4). A longer buildup time requires proportionally longer recycle delays, hence negating any sensitivity gain per unit time. Another consideration is the inhomogeneous hyperpolarization of this sample, with a preferentially higher enhancement for carbohydrate peaks relative to aromatic signals. It is a sign that the radicals were unevenly distributed across different polymer domains, and this spectrum may not accurately reflect the composition.

## 2D ^13^C correlation spectra of unlabeled A. Fumigatus in solid and liquid media

**Fig. 3:**
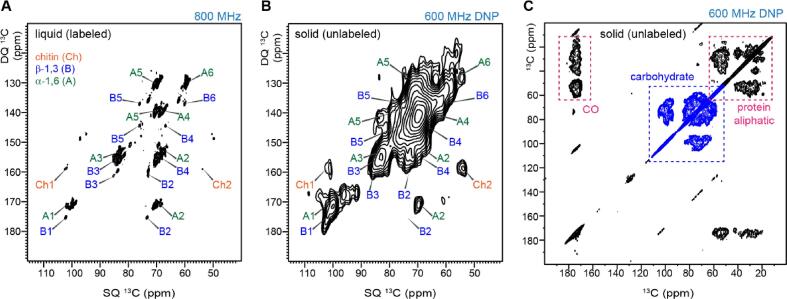
**2D ^13^C–^13^C correlation spectra of unlabeled *A. fumigatus* samples. (A)** CP refocused J-INADEQUATE spectrum measured on 800 MHz NMR at room temperature using ^13^C/^15^N-labeled *A. fumigatus* sample prepared in liquid culture (hyphae). **(B)** CP refocused dipolar-INADEQUATE spectrum of unlabeled *A. fumigatus* solid culture (mostly conidia) measured on 600 MHz/395 GHz DNP. **(C)** DQF 2D dipolar ^13^C–^13^C correlation spectra of unlabeled *A. fumigatus* solid culture sample measured on 600 MHz/395 GHz DNP.

The enhancement of NMR sensitivity gained by MAS-DNP allowed us to collect 2D ^13^C–^13^C correlation spectra using unlabeled *A. fumigatus* materials harvested from the solid media, which is a mixture of conidia and mycelia. In this study, the greenish conidia region was used for NMR measurements. Chemical analysis has reported that the core polysaccharides are conserved (though with variable composition) in the cell walls of hypha and conidium, but the conidium has additional layers of rodlets and melanin on the cell surface while the hypha is covered by galactosaminogalactan (Latgé and Chamilos, 2019). The chemical similarity of carbohydrate structure is informed by the similar spectral patterns of *A. fumigatus* materials cultured in liquid and solid media, providing an implication of the similarity in hyphae and conidia (Fig. 3A, B). Most of the carbohydrate signals observed in the ^13^C-labeled samples of *A. fumigatus* liquid culture, which is hyphae-dominant, showed corresponding signals in the natural-abundance 2D ^13^C–^13^C correlation MAS-DNP spectrum of the solid culture (Fig. 3B).

The chitin signals became stronger in the solid culture, as exemplified by the well-resolved peaks of chitin carbons 1 and 2 (Ch1 and Ch2) that resonate at 103 ppm and 55 ppm on the single-quantum (SQ) chemical shift dimension. This is an indication of a larger amount of chitin in the samples prepared using solid media. Chitin is among the most rigid molecules of *A. fumigatus* cell walls and its signals be preferentially detected by CP-based experiments at room-temperature. However, chitin signals are very weak in the CP refocused INADEQUATE spectrum of liquid culture (Fig. 3A), consistent with its low intensities observed in two recent studies of *A. fumigatus*, which is likely due to the low content of this molecule in liquid cultures (Kang, 2018, Chakraborty, 2021). Signals were also observed for another two other major carbohydrates, α-1,3-glucans and β-1,3-glucans. The results suggest that at least the major constituents are shared between the hyphae and conidia. Many additional signals showed up in the DNP spectrum of the solid culture, likely from more mobile molecules, for example, galactomannan. Mobile carbohydrates are undetectable in the CP-based spectrum at room-temperature (Fig. 3A) but will become visible at the cryogenic temperature of MAS-DNP (Fig. 3B).

Unfortunately, the NMR linewidth, given by the full width at half maximum (FWHM), increased substantially at MAS-DNP conditions. The FWHM linewidths of resolved peaks in this 2D refocused INADEQUATE spectrum were 2–3 ppm. This is considerably broader than the 0.5–0.9 ppm reported for samples measured at ambient temperature at a higher magnetic field (800 MHz) and is worse than the expectation for a spectrum collected on a 600 MHz ss NMR at room temperature. The spectral quality presented in Fig. 3B is much worse than that of unlabeled plant cell walls (Zhao et al., 2020, Kirui et al., 2019). This is due to the more dynamic nature of fungal cell walls compared to their counterparts in plants (Kang, 2018, Zhao et al., 2020). Crystalline components efficiently retain their sharp linewidth at 100 K during MAS-DNP measurements, which has been shown using the cellulose microfibrils in plant biomass (Zhao et al., 2020, Kirui et al., 2019). The major crystalline molecule in fungi is chitin, and it accounts for only 10–20 % of the dry mass of *A. fumigatus* cell wall (Lee and Sheppard, 2016). All other molecules, such as glucans, mannans, and exopolysaccharides, will suffer from the line-broadening effect due to their intrinsic disorder.

The most promising spectrum of unlabeled *A. fumigatus* was collected using a double-quantum filtered (DQF) 2D dipolar ^13^C–^13^C correlation scheme (Fig. 3C) (Wi et al., 2022). This experiment was finished in 30 hrs. The DQF-DARR spectrum of the solid *A. fumigatus* culture showed carbohydrate signals at 60–105 ppm, as well as unexpectedly strong signals of proteins, including both aliphatic carbons (0–70 ppm) and carbonyl groups (165–180 ppm). The quality of the DQF-DARR spectrum is manifestly superior to that of the CP refocused dipolar-INADEQUATE spectrum, considering both resolution and sensitivity as well as simultaneous detection of proteins and carbohydrates. In addition, the dipolar-INADEQUATE spectra collected at natural abundance often exhibit unmatched intensities for two carbons in a spin pair, such as the B1-B2 pair in Fig. 3B, which is not an issue in the DQF-DARR spectrum.

**Fig. 4:**
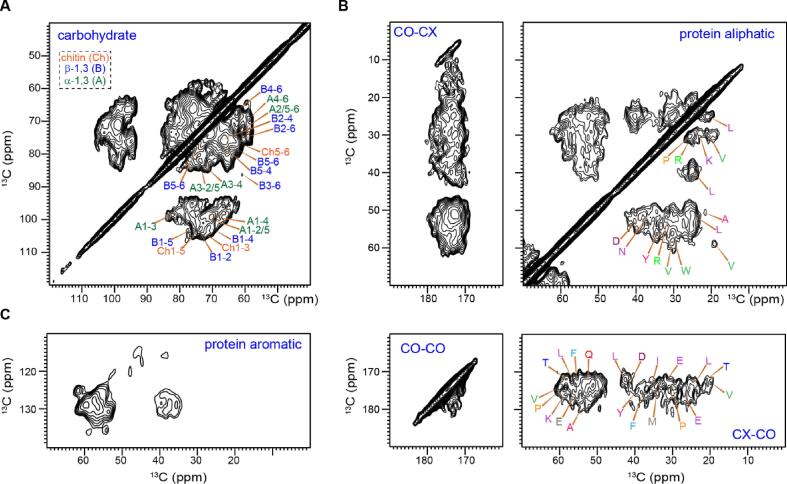
**DNP-enabled 2D correlation spectra of unlabeled *A. fumigatus* solid culture (mostly conidia).** Selected regions of DQF 2D dipolar ^13^C–^13^C correlation spectra are shown for **(A)** carbohydrates, **(B)** protein aliphatic and carbonyl signals, and **(C)** aromatics. The CO presents the carbonyl group in proteins and the CX represents protein aliphatic carbons such as Cα and Cβ. CO-CX refers to the correlation between these carbon sites. The spectrum was acquired using the DQF-DARR sequence on a 600 MHz/395 GHz DNP.

The DQF-DARR spectrum also exhibited satisfactory resolution, allowing us to observe signals from chitin and glucans (Fig. 4A). In addition to many one-bond correlations, the use of 100–250 ms DARR mixing enabled the detection of many medium-range cross peaks. An example is the C1-C3 cross peak of α-1,3-glucan (denoted as A1-3), which showed up at a unique position of (101 ppm, 84 ppm). In addition, the adequate sensitivity allowed us to assign the protein signals to different amino acid types by tracking the correlations among the Cα, Cβ, and CO (Fig. 4B). The chemical shifts are tabulated in Supplementary Table S5. Notably, some cross peaks between the aromatic carbons and the Cα and Cβ were also identified for aromatic amino acids (Fig. 4C). The performance of DQF-DARR is better than most other 2D ^13^C–^13^C correlation experiments, such as the refocused dipolar-INADEQUATE and CHHC that have been frequently employed to investigate unlabeled biomaterials (Viger-Gravel, 2019, Perras, 2017, Kirui et al., 2019, Zhao et al., 2021). This DQF-DARR experiment might be critical to elucidating unresolved structural aspects in fungal cell walls.

## Examination of biopolymer dynamics at room temperature

The dynamics of biopolymers in unlabeled *A. fumigatus* materials were probed using ^13^C-*T*_1_ relaxation, which was mapped using a series of 1D ^13^C spectra with a gradually increasing z-period. This ss NMR approach has been widely used in the field of polymer research, where the samples are typically difficult to label. The information on polysaccharide dynamics afforded by this method perfectly complements the insight about polysaccharide structure obtained using natural-abundance MAS-DNP, as recently demonstrated on rice stems (Zhao et al., 2021).

**Fig. 5:**
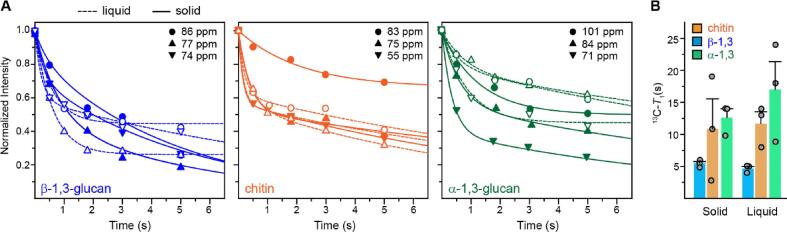
**Dynamics of biopolymers in unlabeled *A. fumigatus* cell walls measured at room temperature. (A)** Representative ^13^C-*T*_1_ relaxation curves of unlabeled *A. fumigatus* samples for β-1,3-glucans (left; blue), chitin (middle; orange), and α-1,3-glucans (right; green). The data of *A. fumigatus* grown in liquid cultures (hyphae) are plotted using open symbols and dash lines. The data of materials prepared in solid media (mainly conidia) are plotted using filled symbols and solid lines. The chemical shifts of key carbon peaks are labeled. **(B)**^13^C-*T*_1_ time constants of different carbon sites in *A. fumigatus* samples prepared in solid and liquid media. The data are fit using bi-exponential equation, plotting only the long (or slow) component of ^13^C-*T*_1_. Data are mean ± s.e.; data points are overlayed on the corresponding bar. The carbon sites and corresponding relaxation time constants are provided in Supplementary Table S6. (For interpretation of the references to colour in this figure legend, the reader is referred to the web version of this article.)

Consistently observed in both the liquid and solid cultures, β-1,3-glucans have the fastest ^13^C-*T*_1_ relaxation (Fig. 5A), thus remaining the most mobile molecule in the cell walls, regardless of the fraction of hyphae and conidia. In contrast, α-1,3-glucans have the slowest relaxation, indicative of its immobility in *A. fumigatus* cell walls. The dynamics of chitin is between the α- and β-1,3-glucans, being intermediately rigid. It should be noted that one carbon site of chitin, the C4 at 83 ppm, has exhibited very slow relaxation that is outside the range of the other carbons. This only happened to the sample prepared using solid media. We suspect that it is due to the insufficient resolution in 1D ^13^C spectra, where the chitin C4 is influenced by the neighboring peak of α-1,3-glucan C3, which is just 1 ppm apart.

The bi-exponential feature became more pronounced in our unlabeled fungal materials (Fig. 5A) compared to the data collected on uniformly ^13^C-labeled samples (Kang, 2018, Chakraborty, 2021). This is because the magnetization exchange between a pair of ^13^C spins, mediated by ^13^C -^13^C spin interactions, gives rise to additional relaxation pathways, thus speeding up the relaxation process, which is no longer efficient at natural ^13^C abundance. The inefficient exchange with mobile motifs also accounts for the substantially longer ^13^C-*T*_1_ time constants for the current unlabeled materials, than the uniformly ^13^C-labeled samples used in recent studies (Kang, 2018, Chakraborty, 2021).

The dynamics can be better analyzed by comparing the ^13^C-*T*_1_ derived from the slow-relaxing component, corresponding to the less mobile domain of the biopolymer (Fig. 5B). The trends observed visually in Fig. 5A still hold. In each sample, the ^13^C-*T*_1_ time constants decreased successively for α-1,3-glucans, chitin, and then β-1,3-glucan. For example, the ^13^C-*T*_1_ time constants of these three molecules in the solid media were 13 ± 1 s, 11 ± 5 s, and 5.5 ± 0.3 s, respectively (Fig. 5B). On the other hand, ^13^C-*T*_1_ was similar in both the solid (5.5 ± 0.3 s) and liquid (5.0 ± 0.3 s) samples for β-1,3-glucans. This recurred for chitin molecules in different samples: the ^13^C-*T*_1_ remained similar in both solid and liquid cultures. In contrast, the ^13^C-*T*_1_ time constant of α-1,3-glucan decreased slightly from 17 ± 4 s in the liquid culture (hyphae) to 13 ± 1 s in the solid culture (mainly conidia). Therefore, α-1,3-glucans are slightly more dynamic in the conidia, though remaining the most rigid molecule across the cell wall.

## Insight into the molecular organization of A. Fumigatus cell walls

It is generally accepted that the chitin molecule, due to its partial crystallinity, should be lending structural support to the fungal cell walls. This mechanical role is reminiscent of the function of cellulose in plant materials, consistent with its slow relaxation observed here. β-1,3-glucans are the major cross-linking polysaccharides in *A. fumigatus* cell walls and are the key components for forming the chitin-β-1,3-glucan-mannan core, a domain containing three covalently linked polysaccharides (Latgé, 2007). A branched analog, β-1,3/1,6-glucan, could also be introduced via the branching site of 3,6-linked glucose residue. With these considerations, it is not surprising that β-1,3-glucan stays as a relatively mobile polysaccharide to maintain its function of crosslinking and branching. The role and dynamics of β-1,3-glucan in the fungal cell wall are similar to those of hemicellulose and pectin (together, named matrix polysaccharides) in the primary plant cell walls (Zhao et al., 2020).

For decades, α-1,3-glucan has remained a mysterious molecule in *A. fumigatus* cell walls. It is largely extractable by hot alkali, thus lacking covalent bonds to other components, which has led to the assumption that α-1,3-glucan is less important to the cell wall organization (Bernard and Latge, 2001, Latgé, 2007). This chemical view has been revamped recently, where a large number of cross peaks (physical contact on the sub-nanometer scale) were identified between chitin and α-1,3-glucan. The ss NMR data support a model in which chitin and the majority of α-1,3-glucan are tightly associated to form rigid aggregates that exclude water molecules to a large extent (Kang, 2018, Chakraborty, 2021). Though this paradigm has come under scrutiny (Kang, 2018, Chakraborty, 2021, Latgé and Wang, 2022), the extremely slow ^13^C-*T*_1_ relaxation of α-1,3-glucan observed in both liquid and solid cultures, with the latter being more relevant to the conditions used in most microbiology studies (Kang et al., 2021, Gastebois et al., 2009, Ashton and Dyer, 2019), confirms the stiffness of most α-1,3-glucans in *A. fumigatus* cell walls.

## An exploratory investigation of unlabeled *C. albicans* samples

**Fig. 6:**
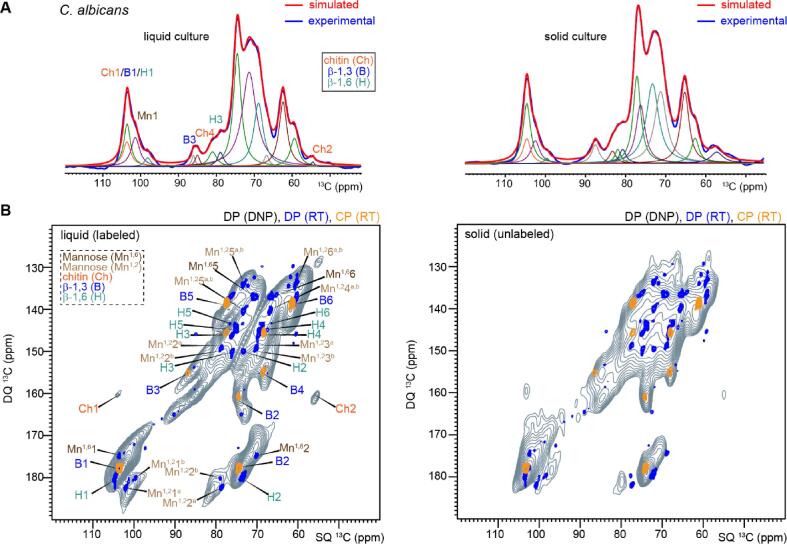
**DNP measurement of *C. albicans* samples. (A)** Spectral deconvolution of 1D ^13^C CP spectra measured on *C. albicans* samples prepared in liquid cultures (left) and solid media (right). The spectra are measured on a 600 MHz/395 GHz DNP system. The simulated spectra (red) match well with the experimentally measured spectra (blue). Underneath are many individual lines that add up to the simulated spectra. (B) 2D ^13^C refocused INADEQUATE spectra of *C. albicans*. The left panel shows the CP refocused dipolar-INADEQUATE spectrum of unlabeled liquid culture measured using 600 MHz/395 GHz DNP (black grey) and the right panel shows the same type of spectrum collected on unlabeled *C. albicans* materials grown in solid media. For each panel, the DNP spectrum is overlaid with two high-resolution 2D spectra measured on ^13^C-labeled liquid culture at room temperature (RT), including a DP refocused J-INADEQUATE spectrum measured on an 800 MHz NMR (blue) and a CP refocused dipolar-INADEQUATE spectrum measured on a 400 MHz ss NMR (yellow). (For interpretation of the references to colour in this figure legend, the reader is referred to the web version of this article.)

We further applied the natural-abundance MAS-DNP method to examine unlabeled *C. albicans* samples. Satisfactory enhancement factors of 26- and 30-fold were obtained from two samples harvested from liquid and solid media, respectively (spectra not shown). Based on the sensitivity, we managed to collect nearly noiseless 1D ^13^C spectra (Fig. 6A), which showed high similarity between the solid and liquid cultures as evidenced by their consistent spectral patterns. This leads to the inference that the growing conditions have relatively minor effect on the composition of cell walls. Spectral deconvolution allowed us to disentangle the underlying carbohydrate resonances (Fig. 6A), and this process is assisted by the resolvable carbon sites in the high-resolution 2D ^13^C–^13^C correlation spectra enabled by MAS-DNP (Fig. 6B). To facilitate the assignment, the 2D ^13^C correlation DNP spectrum of unlabeled *C. albicans* was overlaid with a high-resolution spectrum collected at ambient temperature on a ^13^C-labeled sample (Fig. 6B). In the liquid culture, this comparison allowed us to resolve the signals from β-1,3-glucans, β-1,6-glucans, chitin, as well as some mannose units, which likely originate from peptidomannans in *C. albicans* cell walls (Hall and Gow, 2013). Consistent with the 1D spectra, the 2D spectra also showed a high level of consistency between the cell walls from solid and liquid cultures. Though the spectral resolution is still limited, these observations constitute an early insight into the structural similarity of the core polysaccharides in the yeast form and hyphae of *C. albicans*, and demonstrate the feasibility of using MAS-DNP to investigate different fungal strains. Notably, the *C. albicans* cells mainly exist in the unicellular form (yeast form) observed here. The fungal cell walls, especially those of *C. albicans*, are substantially more mobile than the plant materials and are considered unfavorable for MAS-DNP. The success of natural-abundance DNP thus opens the frontier to interrogate other cellular systems with similar dynamical characteristics, such as microalgae, bacteria, and human and animal cells (Takahashi, 2013, Overall and Barnes, 2021, Albert et al., 2018, Romaniuk and Cegelski, 2015, Poulhazan, 2021, Poulhazan et al., 2018, Arnold et al., 2018, Ghosh et al., 2021), without isotopic enrichment.

## Conclusions

We have shown that the MAS-DNP enabled 2D ^13^C–^13^C correlation experiments of unlabeled fungal materials provide a way for addressing important structural questions that would remain unanswered otherwise. The fungal cell wall may however not be deemed to be the most ideal system for MAS-DNP due to the highly dynamic nature of most fungal carbohydrates (in contrast to plant cell walls) and the significant line-broadening at cryogenic temperature. Despite the difficulty, this strategy has led us to show the similarity of the major polysaccharides in unlabeled fungal materials prepared from solid and liquid media, which could not be possible without MAS-DNP. The carbohydrate fingerprints are also found to be consistent in the conidia and hyphae of *A. fumigatus.* Still, more experiments, especially those designed to measure intermolecular packing, are needed for further assessing the difference in the nanoscale organization. Such development could pave the way for investigating fungal materials that are difficult to label or replicate in the lab, such as disease-relevant fungal isolates and those requiring coculture with human and animal cells.

## CRediT authorship contribution statement

**Liyanage D. Fernando:** Writing – original draft. **Malitha C. Dickwella Widanage:** Writing – original draft. **S. Chandra Shekar:** Writing – review & editing. **Frederic Mentink-Vigier:** Writing – review & editing. **Ping Wang:** Writing – review & editing. **Sungsool Wi:** Methodology. **Tuo Wang:** Conceptualization, Writing – review & editing.

## Declaration of Competing Interest

The authors declare that they have no known competing financial interests or personal relationships that could have appeared to influence the work reported in this paper.
