# Supplementary material for: Solid-state NMR analysis of unlabeled fungal cell walls from Aspergillus and Candida species
Source: J Struct Biol X. 2022 Jul 19;6:100070. doi: 10.1016/j.yjsbx.2022.100070 (PMC9310124; doi:10.1016/j.yjsbx.2022.100070)
Supplement: Supplementary data 1 [file mmc1.pdf]

Supplementary Information

Solid-State NMR Analysis of Unlabeled Fungal Cell Walls from *Aspergillus* and *Candida* Species

Liyanage D Fernando[1#], Malitha C. Dickwella Widanage[1#], S Chandra Shekar[1], Frederic Mentink-Vigier[2], Ping Wang[3], Sungsool Wi[2], and Tuo Wang[1*]

[1] Department of Chemistry, Louisiana State University, Baton Rouge, LA 70803, USA

[2] National High Magnetic Field Laboratory, Tallahassee, FL 32310, USA

[3] Department of Microbiology, Immunology, and Parasitology, Louisiana State University Health Sciences Center, New Orleans, LA 70112, USA

[#] These authors contributed equally

*Correspondence: Tuo Wang (tuowang@lsu.edu)

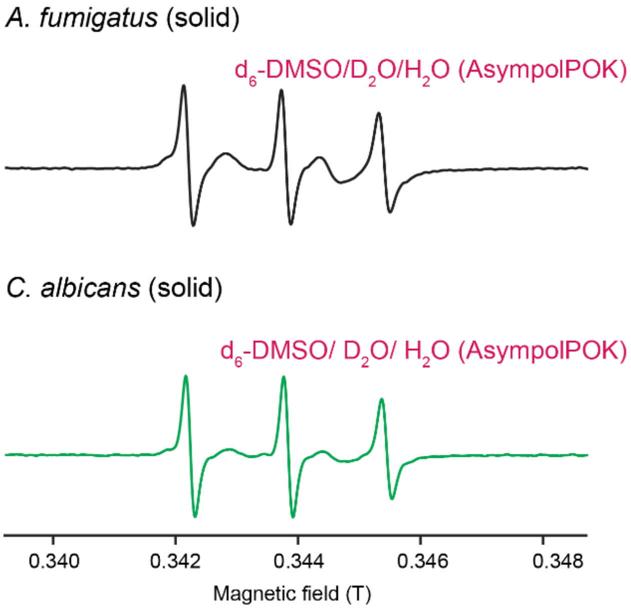

Supplementary Figure 1. Room-temperature EPR spectra of AsympolPok at 9.6 GHz.

Table S1. Experimental parameters used for MAS-DNP and ssNMR on intact fungal cells. Abbreviations are used for ^{1}H Lamour frequency ($\omega_{0,\ 1H}$), total experimental time (t), recycle delay (d1), number of scans (NS), number of points in F2 (td2), number of increments in F1 (td1), acquisition time during t2 for F2 (aq2), and indirect acquisition time for F1 (aq1). Not appliable (-). Experiments were collected on either a 600 MHz/395 GHz MAS-DNP, an 800 MHz ssNMR, or a 400 MHz ssNMR.

Sample	Experiment	$\omega_{0,\ 1H}$ (MHz)	t (hr)	MAS (kHz)	NS	d1 (s)	td2	td1	aq2 (ms)	aq1 (ms)
C. albicans (solid)	1D ^{1}H (MW on/off)	600	2.2	8	2	1.0	-	4096	-	5.7
	1D ^{13}C (MW on/off)		63.7	10.5	64	3.5	-	1024	-	5.7
	1D CP		254		256	3.5	-	1024	-	5.7
	CP refocused dipolar (SPC5) INADEQUATE		20		256	3.5	2048	80	17.2	1.2
C. albicans (liquid)	1D ^{1}H (MW on/off)	600	2.2	8	2	1.0	-	4096	-	5.7
	1D ^{13}C (MW on/off)		72.8	10.5	64	4.0	-	1024	-	5.7
	CP refocused dipolar (SPC5) INADEQUATE		5.6		256	4.0	2048	80	17.2	1.2
	DP refocused J-INADEUQATE	800	0.4	13.5	8	1.5	2600	120	19.4	1.2
	CP refocused dipolar (SPC5) INADEQAUTE	400	5.2	7.5	112	1.7	1800	100	18.0	6.2
A. fumigatus (solid)	1D ^{1}H (MW on/off)	600	2.2	8	2	1.0	-	4096	-	5.7
	1D ^{13}C (MW on/off)		27.3	10.5	64	1.5	-	1024	-	5.7
	^{13}C-T_1	400	120	14	10-40k	2.0	-	1800	-	18.0
	CP refocused dipolar (SPC5) INADEQUATE	600	18.4	10.5	1024	1.3	2048	50	17.2	0.8
	DQF-DARR	600	30	10.5	32/64	5.0	2048	200-300	17.2	0.3
A. fumigatus (liquid)	CP J-INADEQUATE	800	9	13.5	16	2.0	2400	1024	17.9	10.2
	^{13}C-T_1	400	120	14	24-40k	2.0	-	1800	-	18.0

Table S2. Parameters used for the fit of ^{13}C MultiCP spectra in Figure 6A. unk: unknown or unresolved peaks. The error was estimated using the Monte Carlo error estimation of Dmfit.

Δ (ppm)	assignment	amplitude	width (ppm)	integral (%)
		C. albicans liquid culture		
103.1	B1	7511	1.22±0.03	5.7±0.2
85.9	B3	8000	1.05±0.03	2.26±0.07
78.1	B5	8196	1.4±0.1	1.2±0.2
74.4	B2	8305	0.83±0.01	20.1±0.3
68.6	B4	8466	0.56±0.02	18±2
103.4	Ch1	7482	1.43±0.03	3.9±0.2
82.9	Ch3	8115	0.88±0.07	2.1±0.2
54.3	Ch2	8861	1.54±0.07	0.54±0.06
101.8	Mn[1,2]1[a]	7564	0.83±0.02	4.9±0.2
79.6	Mn[1,2]2 [a]	8161	1.11±0.09	1.5±0.2
70.5	Mn[1,2]3 [a]	84116	0.66±0.03	13±2
98.0	Mn[1,2]1[b]	7651	1.05±0.07	1.33±0.09
72.1	Mn[1,6]	8365	0.73±0.03	9±2
62.3	unk	8642	0.82±0.02	10.9±0.2
59.26	unk	8725	0.80±0.02	5.0±0.2
		C.albicans solid culture		
103.1	B1	7478	0.9±0.4	4.3±0.5
85.9	B3	8002	0.63±0.03	2.3±0.1
78.1	B5	8188	0.64±0.06	2.1±0.5
74.4	B2	8305	0.53±0.01	21.1±0.5
68.6	B4	8509	0.62±0.09	4±1
103.4	Ch1	7514	0.95±0.03	4.3±0.4
82.9	Ch3	8130	0.55±0.04	3.4±0.5
54.3	Ch2	8775	0.11±0.01	2.5±0.1
101.8	Mn[1,2]1[a]	7570	0.74±0.03	3.4±0.2
69	Mn[1,2]2 [a]	8453	0.51±0.03	12±1
70.5	Mn[1,2]3 [a]	8397	0.44±0.02	17±2
98.0	Mn[1,2]1[b]	7646	0.89±0.08	1.1±0.2
62.3	unk	8641	0.56±0.08	11±4
59.26	unk	8715	0.8±0.1	1.8±0.5

Table S3. The average cell wall thickness of *A. fumigatus* and *C. albicans*. Results describe the average of 10 measurements from 15 biological replicates of each solid and liquid cultures.

Sample	Cell wall thickness (nm)	
	A. fumigatus	*C albicans*
Solid culture	134±21	195±50
Liquid culture	159±35	182±68

Table S4. DNP buildup time and enhancement factor. The enhancement factor ($\varepsilon_{on/off}$) is obtained by comparing the intensity of spectra measured with microwave on and off. The concentration of biradicals, the culture condition (solid or liquid media), and the composition of the DNP juice (matrix) are also listed.

Sample	Radical	Matrix	DNP build-up time (s)	$\varepsilon_{on/off}$
A. fumigatus (solid)	20 mM c-AsymPol-POK	d_6-DMSO-D_2O-H_2O (1:8:1)	1.3	26
A. fumigatus (solid)	10 mM AMUPol,	d_8-glycerol/D_2O/H_2O (6:3:1)	5.0	90
C. albicans (solid)	20 mM c-AsymPol-POK	d_6-DMSO-D_2O-H_2O (1:8:1)	2.6	30
C. albicans (liquid)			3.1	26

Table S5. **^{13}C chemical shifts of polysaccharides and proteins in fungal cell walls.** The units are in ppm. (/) unidentified. (-) not applicable.

Carbohydrate	Chemical shifts (ppm)								References
	C1	C2	C3	C4	C5	C6	CO	CH_3	
β-1,3-glucan	103.6	74.2	87.5	68.3	77.2	61.5	-	-	Chakraborty et al. 2021[1]
α-1,3-glucan	101	71.9	84.5	69.5	71.7	60.5	-	-	Chakraborty et al. 2021[1]
β-1,6-glucan	103.9	74.6	76.6	70.6	75.7	69.6	-	-	Lowman et al. 2011[2]
Chitin	103.3	55.5	72.9	83.0	75.7	60.9	174.8	22.6	Chakraborty et al[1].
α-1,2-Mannose[a]	101.4	79.2	71.0	67.8	74.1	61.9	-	-	
α-1,2-Mannose[b]	99.1	79.5	71.4	67.8	74.1	61.9	-	-	
α-1,6-Mannose	102.9	71.1	/	/	72.0	66.6	-	-	
Amino Acids	Cα	Cβ	Cγ				CO		
Alanine	52.4	16.3					177.0		Fritzsching et al. 2013[3]
Aspartic acid	54.8	38.3	/				175.9		
Glutamic acid	54.6	28.1	34.1				174.0		
Phenyl alanine	55.5	37.7	/				172.8		
Iso leucine	58.7	36.6	25.1	/	/		173.5		
Lysine	54.2	30.8	22.8		/	/	175.0		
Leucine	52.7	40.3	24.8		/	/	174.4		
Methionine	53.2	31.1	29.9			/	173.4		
Asparagine	51.1	36.6	/				172.6		
Proline	61.1	30.0	25.2				174.2		
Glutamine	53.7	27.4	31.7				173.4		
Arginine	53.9	28.7	25.0				173.7		
Threonine	59.5	67.4	/				172.4		
Valine	59.7	30.7	19.2	/			173.4		
Tryptophan	55.0	28.0	/	/	/	/	173.0		
Tyrosine	58.6	36.0	/	/	/	/	173.0		

Table S6. **^{13}C-T_1 relaxation times of polysaccharides.** The data are fit using bi-exponential equations: $I(t) = e^{\frac{-t}{T_{1b}}}$ and $I(t) = Ae^{\frac{-t}{T_{1a}}} + (1 - A)e^{\frac{-t}{T_{1b}}}$, where A is a perfector. Error bars are standard deviations of fit parameters.

A. fumigatus (liquid culture)					
Assignment	^{13}C (ppm)	A	T_{1a} (s)	B	T_{1b} (s)
B3	86	70 %	0.6 ± 0.1	30 %	5 ± 3
B5	77	40 %	0.4 ± 0.1	60 %	4 ± 2
B2	74	20 %	0.01 ± 0.01	80%	5 ± 1
Ch4	83	28 %	0.20 ± 0.01	72%	14 ± 4
Ch5	75	50 %	0.0045 ± 0.0001	50 %	7 ± 2
Ch2	55	43%	0.17 ±0.06	57 %	13 ± 2
A1	101	20 %	0.5 ± 0.2	80 %	18 ± 6
A3	84	23 %	0.8 ± 0.2	77 %	23 ± 10
A2/5	71	23 %	0.0010 ± 0.0005	77 %	8 ± 2
A. fumigatus (solid culture)					
B3	86	10 %	0.20 ± 0.02	90 %	5 ± 1
B5	77	50 %	0.8 ± 0.3	50%	6 ± 3
B2	72	20 %	0.300 ± 0.001	80%	5.420 ± 0.006
B4	68	20 %	0.0003 ± 0.0001	80%	3.8 ± 0.4
Ch4	83	11%	0.5 ± 0.4	89%	20 ± 6
Ch5	75	11%	0.11 ± 0.05	89%	2.8 ± 0.3
Ch2	55	14 %	0.14 ± 0.01	86 %	10.8 ± 0.3
A1	101	30 %	0.011 ± 0.002	60 %	14 ± 5
A3	84	44 %	0.6 ± 0.1	56 %	14 ± 4
A2/5	71	60%	0.348 ± 0.009	40 %	9.7 ± 0.6

Supplementary References

1. Chakraborty, A.; Fernando, L. D.; Fang, W.; Dickwella Widanage, M. C.; Wei, P.; Jin, C.; Fontaine, T.; Latgé, J.-P.; Wang, T., A molecular vision of fungal cell wall organization by functional genomics and solid-state NMR. *Nat. Commun.* **2021,** *12*, 6346.

2. Lowman, D. W.; West, L. J.; Bearden, D. W.; Wempe, M. F.; Power, T. D.; Ensley, H. E.; Haynes, K.; Williams, D. L.; Kruppa, M. D., New insights into the structure of (1→ 3, 1→ 6)-β-D-glucan side chains in the Candida glabrata cell wall. *PloS One* **2011,** *6*, e27614.

3. Fritzsching, K.; Yang, Y.; Schmidt-Rohr, K.; Hong, M., Practical use of chemical shift databases for protein solid-state NMR: 2D chemical shift maps and amino-acid assignment with secondary-structure information. *J. Biol. NMR* **2013,** *56*, 155-167.
